# Dosimetric Characterization and Workflow Optimization of the FLASH-SARRP for Reliable Preclinical Radiobiological Studies

**DOI:** 10.64898/2026.07.06.736680

**Published:** 2026-07-07

**Authors:** Michèle Knol, Patrik Gonçalves-Jorge, Louis V. Kunz, Pierre Korysko, Benoît Petit, André Durham, Marie-Catherine Vozenin, Pelagia Tsoutsou, Nikolaos Koutsouvelis, Julie Lascaud

**Affiliations:** 1 LiRR - Laboratory of innovation in Radiobiology applied to Radiotherapy/ Faculty of Medicine/ University of Geneva, Geneva, Switzerland.; 2 Radiotherapy and Radiobiology Sector, Radiation Therapy Service, University Hospital of Geneva, Geneva, Switzerland.; 3 University of Oxford, Oxford, United Kingdom.; 4 Department of Medical Physics, Ludwig-Maximilians-Universität München (LMU), Munich, Germany.

**Keywords:** FLASH radiotherapy, FLASH-SARRP, QA workflow, Dosimetric characterization, Scintillating fiber, Preclinical radiobiology studies

## Abstract

**Objective:**

Preclinical small-animal irradiators such as the FLASH-SARRP can support the advancement of photon-FLASH toward the clinic. This study aimed at characterizing the FLASH-SARRP and established a robust quality assurance (QA) workflow to enable accurate and reproducible preclinical experiments.

**Approach:**

Custom 3D-printed spacers were designed to ensure reproducible X-ray tube alignment, sample positioning and mounting of the dosimetric tools. Beam characteristics were evaluated using a combined dosimetric approach. High spatially resolved dose distributions were obtained from Gafchromic films, whereas a plastic scintillating fiber was employed to monitor in real-time the temporal pulse structure and synchronization between the two X-ray tubes. Day-to-day variability of the delivery was evaluated over several sessions.

**Main results:**

The FLASH-SARRP achieved dose-rates of around 80 Gy/s when both tubes were used simultaneously and provided a homogeneous irradiation field suitable for small-animal studies. A desynchronization between the two tubes was observed with an average delay of 10 ms, resulting in temporal dose-rate heterogeneity. Additionally, a substantial inter-session variability (~11%) was found, whereas the intra-session variability was relatively low (~4%). Inter-session variability was reduced to 5%, approaching the intra-session variability, by adding Gafchromic films/scintillator-based quality assurance (QA) workflow into the irradiation routine.

**Significance:**

This work highlights the importance of temporal dosimetry for preclinical FLASH studies. Additionally, a practical QA framework is proposed integrating real-time monitoring with reference dosimetry. The proposed work enables adaptive dose delivery, thereby enhancing the reproducibility of the irradiations, which is crucial for reliable preclinical studies on the FLASH effect.

## Introduction

FLASH radiotherapy is characterized by the delivery of radiation at ultra-high dose-rates (UHDR) and has been shown to reduce normal tissue toxicity while maintaining anti-tumor efficacy compared to radiotherapy at conventional dose-rates, a biological phenomenon called the FLASH effect (Favaudon et al 2014). The FLASH effect has now been demonstrated in various *in vivo* experimental models (Vozenin et al 2022, 2026) with several beam types, including electrons, protons, photons and carbon ions (Vozenin et al 2024). Today, the major challenges in FLASH research are related to accelerator technology, dosimetry and treatment planning system development, as well as to understanding the biological basis of the FLASH effect. Preclinical FLASH research combines both aspects, understanding the mechanisms of the differential effect in normal tissues versus tumors and defining the optimal irradiation parameters required to elicit the FLASH effect (Knol et al 2026).

Further research is needed to advance photon-FLASH beams, as photons are the most widely used type of radiation in the clinic, and therefore the most likely to be largely adopted by the radiation oncology community. Unfortunately, photon FLASH remains technologically the most challenging, as reaching ultra-high dose-rates with bremsstrahlung targets is a significant limitation due to target heating. Nevertheless, the technology is rapidly evolving, and photon-FLASH platforms are becoming available in the US with the PHASER system from TibaRay, Inc. (Maxim et al 2019) and in China with the CHEX system from Zhongjiu Flash Medical Technology Co., Ltd. (Lin et al 2025). Experimental data are now also emerging from photon-FLASH systems. The first report of the FLASH sparing effect induced by photons in the brain was published already in 2018 using a synchrotron (Montay-Gruel et al 2018) and recently more biological results have been made available (Gao et al 2022, Miles et al 2023, Brown et al 2025, Lin et al 2025, Ghita-Pettigrew et al 2026, Zhang et al 2026, Hao et al 2026) stimulated by the development of photon FLASH technological platforms at Hopkins by the group of Wang and Rezaee (Rezaee et al 2021, Miles et al 2023, 2024) and Myanyang by the group of Zhang and Du (Gao et al 2022).

The former is now commercialized by Xstrahl, Inc, called the FLASH-SARRP, and consists of a self-shielded cabinet equipped with two opposing kilovoltage X-ray tubes with rotating anodes, enabling both UHDR as well as conventional irradiations. A commissioning study on the FLASH-SARRP has previously been published (Tajik Mansoury et al 2025), showing linear output with respect to exposure time and tube current, dose-rates up to 100 Gy/s, and a field uniformity within 4 %.

In order to carry out reliable and reproducible biological FLASH experiments, a few essential conditions must be satisfied. The irradiator should be able to reach dose-rates higher than at least 40 Gy/s. To allow partial irradiation of small animals, a homogeneous irradiation field of at least 100 mm^2^ is needed. Moreover, the system should ensure reproducible dose delivery in subsequent irradiations, as well as from day-to-day, as even slight differences in the delivered dose, dose-rate, or pulse structure can alter the biological outcome (Vozenin et al 2020, Tobias Böhlen et al 2024). Therefore, defining a robust quality assurance (QA) workflow is essential to ensure precise and reproducible biological experiments, which is the aim of this present work. Additionally, this QA workflow must account for the specific configuration of the FLASH-SARRP system, which includes two X-ray tubes. This introduces additional complexity in terms of temporal synchronization, geometric alignment between both tubes, and subsequently the homogeneity of the irradiation field.

The aim of this study was, therefore, to establish a QA workflow for the FLASH-SARRP in preparation for biological FLASH experimentation. To this end, we implemented Gafchromic films (Ashland Inc.) and a plastic scintillating fiber (Luxium solutions) into custom-designed spacers to characterize the system under various experimental configurations and to account for the day-to-day variation of the system. While films are well-suited for dose measurements and offer a high spatial resolution (Jaccard et al 2018, Jorge et al 2019), plastic scintillating fibers have a very fast response time in the range of nanoseconds, a linear dose response, near water-equivalence, and can easily be integrated into an experimental setup thanks to their small size and flexibility (Hart et al 2022, Baikalov et al 2025). This workflow will support future experimental studies, initially focusing on biological validation of the FLASH effect to determine acceptable variations in synchronization of the tubes and system stability.

## Methods

### Irradiation Setup

All irradiations were performed with the FLASH-SARRP system using either a single tube or both X-ray tubes simultaneously. In FLASH mode, the tube current has a range of 10–630 mA, and a peak voltage of 150 kVp, with selectable irradiation times between 1 and 250 ms in discrete increments. No external filtration was applied; however, an internal filtration of 0.7 mm aluminium was present within the tubes. External fans were used between irradiations to enhance cooling of the machine and prevent overheating due to the fast rise in temperature of the tubes. The distance between the tubes was minimized (i.e., 7 cm distance between irradiation center and the tube sources) to reach the highest dose-rates achievable with this system.

**Figure 1.**
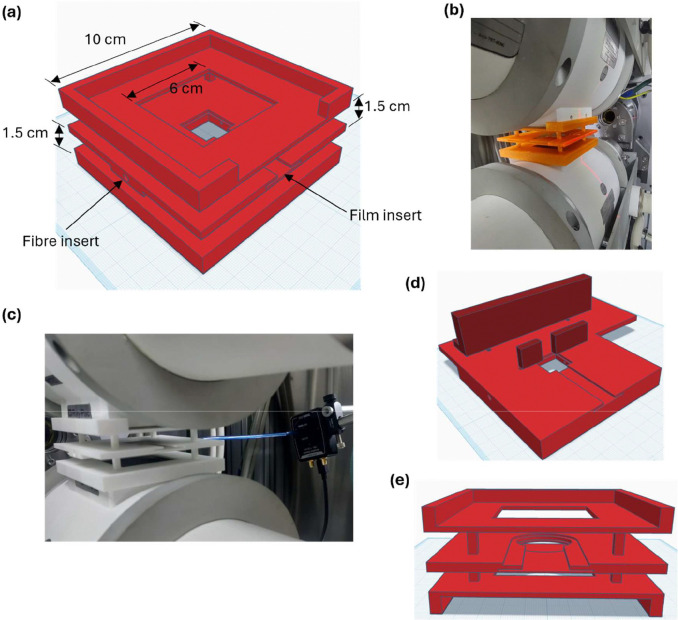
The setup of the film and fibre measurements in the SARRP FLASH. **(a)** 3D model of the spacer including a slit for Gafchromic positioned at a distance of 7 cm from the source of both tubes and a hole for the plastic scintillating fibre directly below the film. Model created in Tinkercad. **(b)** A photo of the spacer setup between the two tubes used for film measurements. **(c)** A photo of the spacer setup with the plastic scintillating fibre and connected to the photodetector. **(d)** 3D model of the spacer used for knee irradiations using only the bottom tube. A slit for Gafchromic film and a hole for the plastic scintillator is implemented in a similar way to the original spacer in A. **(e)** 3D model of the spacer used for Zebrafish embryo irradiations with both tubes.

A set of dedicated 3D-printed spacers was designed to ensure reproducible positioning of the tubes, samples and dosimetry tools across experiments. The base structure of the 3D-printed platform used for the dosimetry experiments is depicted in Fig. 1a–c. The two open extremities are shaped to fit to the tube surface, while an intermediate layer is printed at mid-height with a 6 × 6 mm^2^ central aperture to insert the Gafchromic film and a transverse through-hole to guide the scintillating fiber. The design of the 3D spacer was optimized to minimize interactions with the primary beam. This baseline platform was adapted to fit the needs of future biological experiments, including irradiations of biological samples (e.g., zebrafish embryos) in a Petri dish with both tubes (Fig. 1e), or irradiations of the legs of small animals using only the bottom tube (Fig. 1d). Extra height adjusters were designed to be placed between the printed platform and the tube to enable precise modification of the source-to-surface distance (SSD). All elements were modelled in the open-source Tinkercad software and printed by a BambuLab A1 mini, using either PLA or PETG filaments. A 0.4 mm nozzle, 0.2 mm layer thickness and a standard infill of 15% were used.

### Film Dosimetry

To assess the shape and uniformity of the beam, as well as to obtain precise dose measurements, EBT-XD Gafchromic film was used. The films were calibrated against the SRT-100 (Sensus Healthcare, United States) at the Geneva University Hospitals (HUG), a 100 kV clinical photon irradiator calibrated and traceable to primary standards at the Swiss institute of metrology (METAS). After 24 hours of development in a dark environment, films were scanned with an Epson Expression 10000XL flatbed scanner, using the positive film mode at 300 dpi without further corrections and saved as a tiff file. Files were analyzed with a custom Python script, using the red channel after background subtraction. The background was defined as the average value from an unirradiated film of the same batch.

When both tubes were used, the reported dose was defined as the average value in the homogeneous central part of the field within a circular region of interest (ROI) of 10 mm diameter. When only the bottom tube was used, the homogeneous part of the field was located towards the top of the field, where a circular ROI of 10 mm was used to determine the average dose.

### Depth dose measurement, linearity and field homogeneity assessment

Percentage Depth Dose (PDD) curves of the beam central axis were obtained using 1 cm-thick RW3 solid water slabs (PTW-Freiburg GmbH, Freiburg, Germany) (50×50 mm) stacked between both tubes (Supp. Fig. 1a). Gafchromic films were placed between the solid water slabs and at the surface of both tubes. Irradiations were performed using identical parameters (150 kV, 630 mA, 100 ms) with either one or both tubes.

Dose linearity with exposure time and tube current were evaluated using the spacer from Fig. 1a and Gafchromic film. Films were irradiated subsequently by varying either time or current, while keeping the other parameters constant (150 kV, 630 mA, 100 ms).

The homogeneity of the irradiation field at the foreseen biological sample position (i.e., at intermediate distance between the tubes) was evaluated by irradiating Gafchromic films with the dosimetry spacer shown in Fig. 1a.

Flatness was calculated as $\left(D_{\max}-D_{\min}\right)/\left(D_{\max}+D_{\min}\right)*100\%$ with the central 80% of the field, defined by the 50% isodose lines. Similarly, symmetry was calculated as $max\;(\vee D(x)-D(-x)\vee )/D_{\mathrm{centre}}*100\%$.

Reproducibility was assessed across eight independent sessions spread over several weeks. Both tubes were used at 150 kV, 630 mA, and for a total irradiation time of 125 ms to target a dose of about 10 Gy. Gamma index analysis was performed using criteria of 2 %/2 mm, adapted to the small scale of preclinical irradiations.

### Fiber measurements

To obtain information about the dose variation over time at the pulse scale, a plastic scintillating fiber (BCF-60XL, 1 mm diameter, Luxium solutions) was used. The fiber was mounted to a silicon amplified photodetector with an integrated acquisition system (PDA36AU, Thorlabs). The fiber output signal was amplified by 70 dB and acquired using the provided PDA_USB software at a sampling rate of 1 kHz.

Fiber measurements were analyzed using a custom Python script. The signal was filtered in post-processing by using a moving average and subsequently a zero-phase Butterworth low-pass filter of the fourth order. The start and the end of the pulse were detected by their rising and falling edges using an adaptive threshold set to 20% above the baseline. From this, the total irradiation time was determined, as well as the delay between the tubes, the amplitude, background noise, and the area under the curve (AUC).

Reproducibility measurements were taken during various sessions with only the fiber integrated in the two-tube dosimetry spacer (Fig. 1a), always using the same irradiation parameters (150 kV, 630 mA, 100 ms).

### QA workflow

The proposed QA workflow is divided into two steps. First, a cross-calibration was made between the fiber and the films. To maximize the precision, the calibration was performed on the days before the planned biological experiments, using the same irradiation platform, which was not moved afterwards, and the same irradiation parameters. Both the film and the scintillating fiber were irradiated simultaneously in FLASH mode at the maximum beam current. Irradiations were repeated typically five times for different doses, controlled by varying the irradiation times between 32 and 250 ms. Afterwards, the relationship between the AUC from the fiber signal and the measured dose from the films was determined, resulting in a cross-calibration factor between the AUC and absolute dose.

Second, on the day of the planned experiments, the setup was irradiated several times at the end of the system warmup with the scintillating fiber in place. Instantaneous dose-rate surrogates, in units of AUC/ms, were determined from the average peak power of the scintillator signal and the pulse duration. The average instantaneous dose-rate surrogates were converted to absolute instantaneous dose-rates, in units of Gy/s, based on the previously determined cross-calibration factor. In case of large deviations, the SSD or the pulse duration was adapted to match the requested dose-rate and dose, assuming an inverse square relationship between the deposited dose and its distance from the source.

The robustness of the QA workflow was assessed for single-tube irradiation using the bottom tube and dedicated platform (Fig 1d). Several SSDs were tested in a single session using the height adjusters and multiple irradiation times between 50 and 250 ms were used.

### Statistical Analysis

The intra-session variability was calculated from the reproducibility film measurements. The coefficient of variation (CV) was calculated for each session as the ratio of the standard deviation to the mean of the measured doses. A pooled intra-session CV was then derived from a one-way ANOVA. Inter-session variability was calculated as the standard deviation of the session means divided by the overall average dose. For the validation of the QA workflow, doses were first normalized to the used irradiation time before determining the variation.

## Results

### Spatial dose distribution

**Figure 2.**
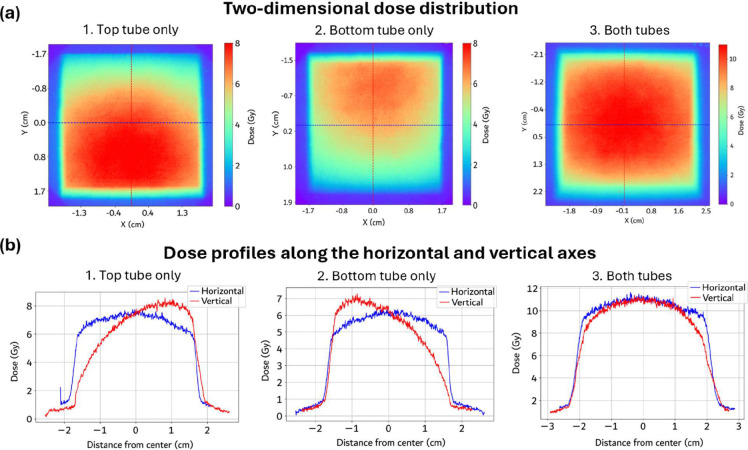
Beam characteristics. **(a)** Dose maps obtained from irradiated Gafchromic film with the top tube only (1), bottom tube only (2), or both tubes (3). The crosshairs through the middle of the field indicate the line used for the dose profiles. **(b)** Dose profiles through the middle of the field from the film irradiated with the top tube only (left), bottom tube only (middle), and both tubes simultaneously (right). The blue line indicates the horizontal line profile, the red line the vertical profile.

The two-dimensional dose depositions obtained when using either only the top tube, only the bottom tube, or both tubes together, are depicted in Fig. 2a, with corresponding dose profiles extracted from the same Gafchromic film measurements in Fig. 2b. For single tube irradiation, profiles were heterogeneous, resulting from the heel effect, going in opposite directions for the two opposing tubes. The most homogeneous region of the field of the top tube is located towards the bottom of the field, while the bottom tube produced a homogeneous dose in the upper portion. When both tubes were operated simultaneously, their individual contributions complement one another, resulting in a centrally homogeneous dose distribution (Fig. 2a, b). Analysis of these dose maps showed an average field size of 4.2 × 4.0 cm, defined by the 50% isodose lines (Table 1). Furthermore, flatness was 9 % horizontally and 14 % vertically, consistent with the vertical asymmetry introduced by the heel effect, while symmetry was comparable across both directions (7%).

Depth dose measurement in FLASH-mode revealed a half-value layer (HVL) of around 14.2 mm water equivalent thickness. When both tubes were operated simultaneously, the dose distribution is homogeneous along the beam direction, with a slightly larger contribution of the top tube.

### Time profiles and tube synchronicity

**Figure 3.**
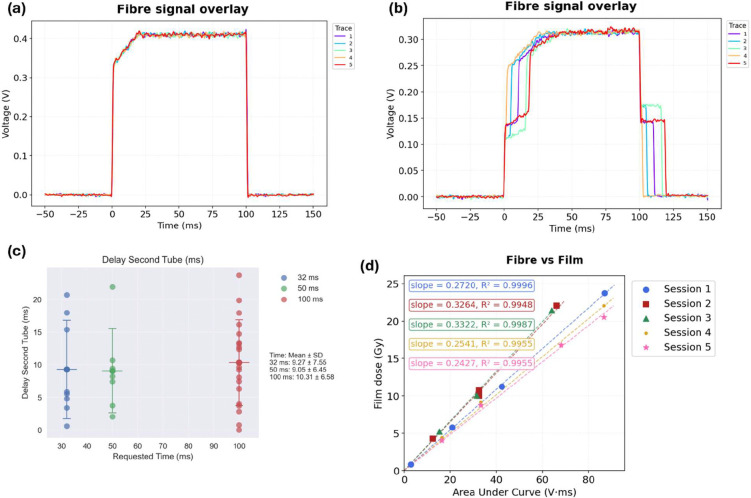
Fibre measurements of the delivery time profile. **(a)** Overlay of 8 irradiations measured during the same session with the same parameters. The smoothed signal is plotted. **(b)** Overlay of 5 irradiations (bottom tube only) measured during the same session with the same parameters. The smoothed signal is plotted. **(c)** The delay of the second tube, measured by the fibre measurements, defined as the time between the start of the first tube and the start of the second tube. Irradiations were performed during various sessions using either 32, 50, or 100 ms. **(d)** The AUC of the fibre measurements and corresponding dose determined from Gafchromic films, together with a linear fit.

The time profile of the X-ray pulse for a single-tube irradiation is presented in Fig. 3a for five deliveries using the bottom tube within the same session. As seen, the fiber output resembles a step-like function with a ramp-up at the start. The pulse structure within the session was highly reproducible in terms of both the duration and amplitude, and therefore, the AUC.

When both tubes were operated simultaneously, fiber measurements revealed a systematic offset between the start of the irradiation of the two tubes (Fig. 3b). Although the overall shape of the two individual X-ray pulses and their requested irradiation time were preserved, they did not fire synchronously. The delay between the onset of the first and the second tube randomly varied between 0 and 24 ms, with a mean delay of 10 ms (Fig. 3c). The delay proved to be independent of the set irradiation time and no consistent pattern was observed as to which tube fired first. Consequently, the total irradiation time was increased, and the instantaneous dose-rate was reduced by a factor of two at the beginning and end of the pulse, introducing temporal dose-rate heterogeneity within a single irradiation delivery.

### Intra- and inter-session variability

**Figure 4.**
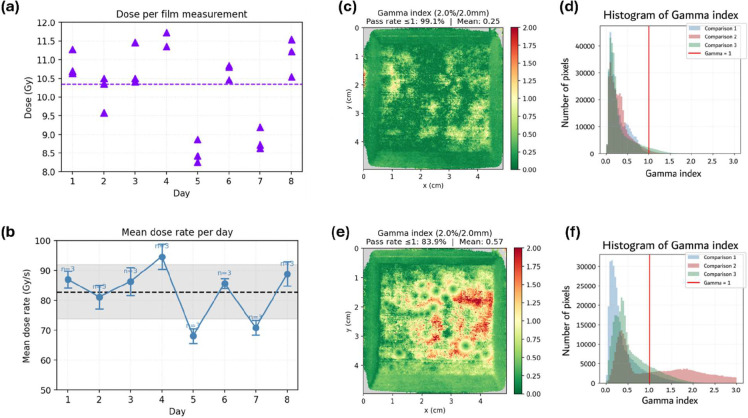
Intra- and inter-session variation of the irradiation field. **(a)** Measurements over several irradiation sessions with both tubes. Dose was determined with Gafchromic film. Irradiations were all carried out with the same parameters (150 kV, 630 mA, 125 ms). The mean of all these measurements is shown with the dashed line (125 ms: 10.4 Gy). **(b)** The dose rate over time determined with the dose from the Gafchromic film and the irradiation time from the measurements from (a). The mean of all measurements is shown with the dashed line (83 Gy/s), the grey area depicts one standard deviation. **(c)** A representative gamma index map (2%/2mm) between dose maps of irradiations performed during the same session. **(d)** An overlay of histograms of the gamma indices of three comparisons of irradiations performed during the same session. **(E)** A representative gamma index map (2%/2mm) between dose maps of irradiations performed during two different sessions with the same parameters. **(f)** An overlay of histograms of the gamma indices of three comparisons of irradiations performed during three different sessions.

Intra- and inter-session variability were assessed by repeated measurements across multiple sessions. The results of the variability assessment are presented in Fig 4. The dose in the homogeneous part of the irradiation field varied between 8.3 and 12.4 Gy during the sessions, with a mean and standard deviation of 10.4±1.2 Gy (Fig. 4a), corresponding to an inter-session variability of 11%. The intra-session variability (4%) was slightly lower for the investigated parameters. This gave rise to dose-rates ranging across sessions from 66 to 99 Gy/s, with an overall average value of 82.8 Gy/s, whereas dose-rate variation within a single session was substantially lower (Fig. 4b).

The gamma index, which simultaneously accounts for spatial and dosimetric differences between irradiations, was used to evaluate the reproducibility of the dose distribution and field geometry. A gamma index below 1 is considered to be acceptable. For intra-session comparisons, a representative gamma index map demonstrated high agreement between irradiations, with a mean gamma index over the whole field of 0.25 and 99.1% of pixels passing the gamma criterion (Fig. 4c). The overlay of multiple intra-session comparisons confirmed this low variability (Fig. 4d). However, for inter-session comparisons a higher mean gamma index over the field of 0.57 was found, with only 83.9% of the pixels passing the threshold (Fig. 4e). Additionally, the overlay of several inter-session comparisons confirmed the substantially larger spread in both the dose magnitude and the field geometry compared to the intra-session results (Fig. 4f). Together, these results indicate significant day-to-day variations of the maximum dose and uniformity of the field that should be accounted for prior to biological experiments to ensure precise and reproducible irradiations.

**Figure 5.**
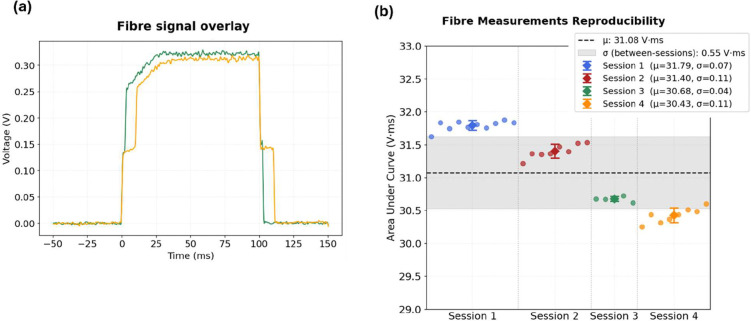
Evaluation of the fibre measurement reproducibility. **(a)** The smoothed signal of 2 measurements (100 ms) taken during different irradiation sessions. **(b)** Integral of the fibre signal (area under the curve, AUC) taken during different irradiation sessions (n = 5–10). All measurements were taken during 100 ms irradiations using both tubes. The mean AUC of all measurements is depicted with the dashed line; the grey area shows 1 standard deviation. The mean and standard deviation are also shown per session.

The reproducibility was also assessed through fiber measurements. Irradiations were carried out over several sessions, using the same irradiation parameters. Overlaying the fiber signals from different sessions revealed a variability in the amplitude of the signal that was not observed during the intra-session comparisons of the fiber measurements, pointing again to the session-dependent output variation and showing that this is reflected in both film and fiber measurements (Fig. 5a). Quantification of the signal by determining the AUC across all fiber measurements confirmed a low intra-session variability of 0.25% and a substantially higher inter-session variability of 1.77% (Fig. 5b).

### Cross-calibration of the scintillating fiber

**Figure 6.**
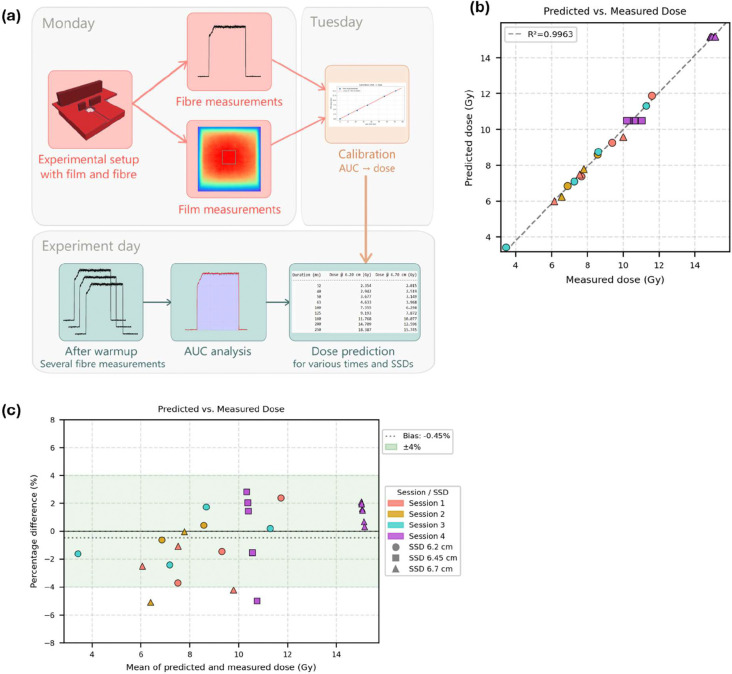
QA framework biological experiments. **(a)** Schematic of the workflow used for biological experiments. **(b)** Predicted versus the measured dose with a linear fit. Doses were measured with Gafchromic films over four different sessions. Doses were predicted according to the workflow presented in a. Measurements were performed using the spacer for knee irradiations as shown in fig. 1d. Colours represent the different sessions; shapes correspond to different SSDs (legend in c). **(c)** The mean of the predicted and measured dose versus the percentage difference. Measurements are the same as in b. The inter-session variability is depicted as the green area.

To be able to relate the signal of the fiber quantitatively to the Gafchromic film data, simultaneous film and fiber irradiations were performed across multiple sessions using both tubes. Comparing fiber to film output across sessions and with various irradiation times demonstrated a linear relationship between the two modalities within a single session (Fig. 6c). However, it should be noted that, with the present irradiation setup, the slope of the obtained calibration curves varied slightly between sessions.

### Assessment of the QA workflow robustness

As the observed day-to-day variation makes it challenging for biological FLASH experiments, where accurate and reproducible dosimetry is absolutely necessary, we designed a QA workflow to account for this variation and reduce it to only the inter-session variation. The predicted dose was in good accordance with the measured dose from the films when this workflow was validated during multiple sessions, showing a linear relationship with an R^2^ of 0.9963 (Fig. 6b). These irradiations were performed using various irradiation times and SSD, thus validating a large range of the predicted doses. The percentage difference between the predicted and the measured dose for these irradiations showed an underestimation of the predicted doses with an average bias of −0.45% (Fig. 6c). Moreover, using our protocol, the day-to-day variation has been reduced to almost that of the intra-session variation (4.7%).

## Discussion

In this work, we characterized the temporal and spatial dose delivery of a photon-FLASH platform by combining the use of a plastic scintillating fiber as a real-time monitoring tool with Gafchromic films for absolute dosimetry. Gafchromic films revealed a dose distribution, field size, and depth dose curve, consistent with previously reported results from similar FLASH-SARRP systems (Tajik Mansoury et al 2025, Miles et al 2024). Meanwhile, the flatness and symmetry values exceeded those typically accepted for clinical irradiators, and they are also slightly higher (7%) than comparable preclinical irradiation systems (~ 3%) (Wang et al 2017, Feddersen et al 2019). However, a homogeneous circle of 1-cm diameter in the center of the field is sufficient for the irradiation of small animals. Together, these results provide a comprehensive spatial characterization of the beam, which is crucial for establishing accurate dosimetry for future preclinical studies.

The implementation of a scintillating fiber in the FLASH-SARRP setup revealed a substantial desynchronization between the two tubes, with an average delay of 10 ms. Such a delay extends the effective total irradiation time beyond the requested total irradiation time; for example, a mean delay of 10 ms on a 100 ms irradiation corresponds to a 10% increase in total irradiation time. This affects the overall dose-rate profile and, consequently, the instantaneous dose-rate, where the first and last 9% of the irradiation will be carried out with half of the dose-rate. Critically, this heterogeneity of the instantaneous dose-rates within one irradiation can influence whether the FLASH effect is reliably achieved (Liu et al 2025, Zhu et al 2025). The desynchronization is an even bigger concern for lower doses, since the delay proved to be independent of the total irradiation time. An average delay of 10 ms on a 32 ms irradiation (corresponding to approximately 3 Gy) will cause a 30% increase of the total irradiation time, as well as the first and last 25% of the irradiation with only half of the dose-rate. With an average dose-rate of around 80 Gy/s, this would correspond to part of the irradiation that will be carried out with only around 40 Gy/s, a dose-rate that might not be sufficient to trigger the FLASH effect. Additionally, since the delay varies substantially, the extent of the change in instantaneous dose-rate will vary from irradiation to irradiation and will thus not be reproducible. This poses an extra problem when fractionated regimens are used, typically with lower doses per irradiation, since not all fractions will be delivered with the same dose-rate, possibly leading to higher chances of losing the sparing effect, and without regulation of the FLASH delivery. At present, the FLASH sparing effect with the FLASH SARRP has only been reported *in vivo* using one tube (Brown et al 2025, Ghita-Pettigrew et al 2026). *In vivo* studies remain to be done using both tubes to see if a sparing effect can reliably be found. In the meantime, a solution needs to be found to decrease the offset significantly.

Comparing the output of the film to that of the fiber confirmed that both detectors capture the same underlying dosimetric variation and support the use of the fiber as a reliable real-time dosimetric monitor, complementary to film-based reference measurements. However, a variation was found in the calibration factor between the two dosimeters. This is attributed to variations in the repositioning of the fiber when remounting the irradiation holder between sessions (e.g., between sessions with one- or two-tube spacer) and is mitigated by systematically performing a new calibration after changing the irradiation platform. This weekly calibration was therefore implemented in the proposed QA workflow.

Both film and fiber measurements demonstrated an acceptable intra-session variability but consistently identified a higher inter-session variability. This increased inter-session variability complicates both the reproducibility and the standardization of FLASH delivery, a prerequisite when assessing subtle biological differences. Importantly, however, the real-time output of the plastic scintillating fiber enables adaptive delivery strategies, whereby the SSD or irradiation time can be adjusted to compensate for the day-to-day output variation, a correction that film-based dosimetry alone cannot support.

This work shows that the integration of a scintillating fiber into the FLASH-SARRP experimental setup can be used as both a characterization and an active QA tool for preclinical FLASH experiments. The characterization indicated significant day-to-day variations of the maximum dose and uniformity of the field that should be accounted for prior to biological experiments, to ensure precise and reproducible irradiations. To this manner, using the fiber as a QA tool can reduce the day-to-day variation to a similar level as the intra-session variation, while providing additional information on the time structure of the beam.

There are, however, several limitations to the proposed scintillator-based dosimetry tool. The scintillation yield has been shown to be temperature-sensitive, with changes between 0.5 and 3% per 5 degrees Celsius of heating (Buranurak et al 2013). Day-to-day variation of the room temperature or heating of the X-ray tubes during long series of irradiation could then directly impact the dosimetry outcomes if not taken into account. The proposed setup would therefore benefit from the integration of a temperature monitoring system for real-time temperature compensation.

A further limitation of the current setup is the sensitivity of the detected scintillation light to fiber positioning. To ease the mounting of the scintillator and irradiation platform within the FLASH-SARRP cabinet, the fiber has to be unmounted every time, and no solid coupling was used between the fiber and the photodetector. Hence, small changes in the angle or distance between the two optical parts from one session to another can affect signal amplitude, contributing to inter-session variability and making it difficult to decouple positioning-induced signal changes from true variations of the machine output. Nevertheless, despite the fiber being repositioned between sessions, variability measured remained comparable to that observed with films, albeit with a slightly different calibration slope. This highlights the importance of performing a dedicated QA in the days prior to biological experiments, after the irradiation platform has been mounted. That way, reference measurements account for mounting error and eliminate this uncertainty. Additionally, in our current setup the fiber was cross-calibrated against film measurements, providing relative dose output only rather than absolute dose measurements. Finally, the robustness of the QA workflow was only validated using the bottom tube due to technical limitations. For a more robust validation, the QA workflow should also be validated using both tubes.

## Conclusion

In this work, we developed and validated a practical and robust dosimetry framework combining Gafchromic films and a plastic scintillating fiber for preclinical FLASH experiments using the FLASH-SARRP. As previously reported, our results show that the FLASH-SARRP system delivers dose-rates and irradiation field homogeneity compatible with preclinical FLASH studies.

Importantly, our study revealed large inter-session variability and tube asynchronicity. While the latter is intrinsic to the irradiator and can only be recorded to provide a more comprehensive understanding of the biological outcomes, the former represents a major source of experimental uncertainty that can compromise reproducibility if not properly addressed.

We demonstrated that this variability was effectively mitigated using the proposed QA workflow for FLASH preclinical experiments, integrating real-time fiber monitoring with film-based dosimetry. This approach improves the reliability and reproducibility of FLASH irradiations by correcting day-to-day output fluctuations, providing a practical framework for standardized preclinical studies.

## Supplementary Material

Supplement 1

## Figures and Tables

**Table 1.** Beam characteristics. Flatness was calculated as $\left(D_{\max}-D_{\min}\right)/\left(D_{\max}+\right.\left.D_{\min}\right)*100\%$ with the central 80% of the field (defined by the 50% isodose lines). Similarly, symmetry was calculated as $max(\vee \;D(x)-D(-x)\vee )/D_{\mathrm{centre}}*100\%$. The mean of 6 different measurements was taken in both the horizontal and the vertical direction. The field width was determined as the size between the 50% isodose lines.

*μ* ± *σ*	Flatness (%)	Symmetry (%)	Field width (cm)
**Horizontal**	9 ± 1	7 ± 1	4.2
**Vertical**	14 ± 1	7 ± 1	4.0
