# Supplementary material for: Dosimetric Characterization and Workflow Optimization of the FLASH-SARRP for Reliable Preclinical Radiobiological Studies
Source: bioRxiv. 2026 Jul 7:2026.07.06.736680. Preprint. [Version 1] doi: 10.64898/2026.07.06.736680 (PMC13370466; doi:10.64898/2026.07.06.736680)
Supplement: Supplement 1 [file NIHPP2026.07.06.736680v1-supplement-1.pdf]

bioRxiv preprint doi: https://doi.org/10.64898/2026.07.06.736680; this version posted July 7, 2026. The copyright holder for this preprint (which was not certified by peer review) is the author/funder, who has granted bioRxiv a license to display the preprint in perpetuity. It is made available under a CC-BY 4.0 International license.

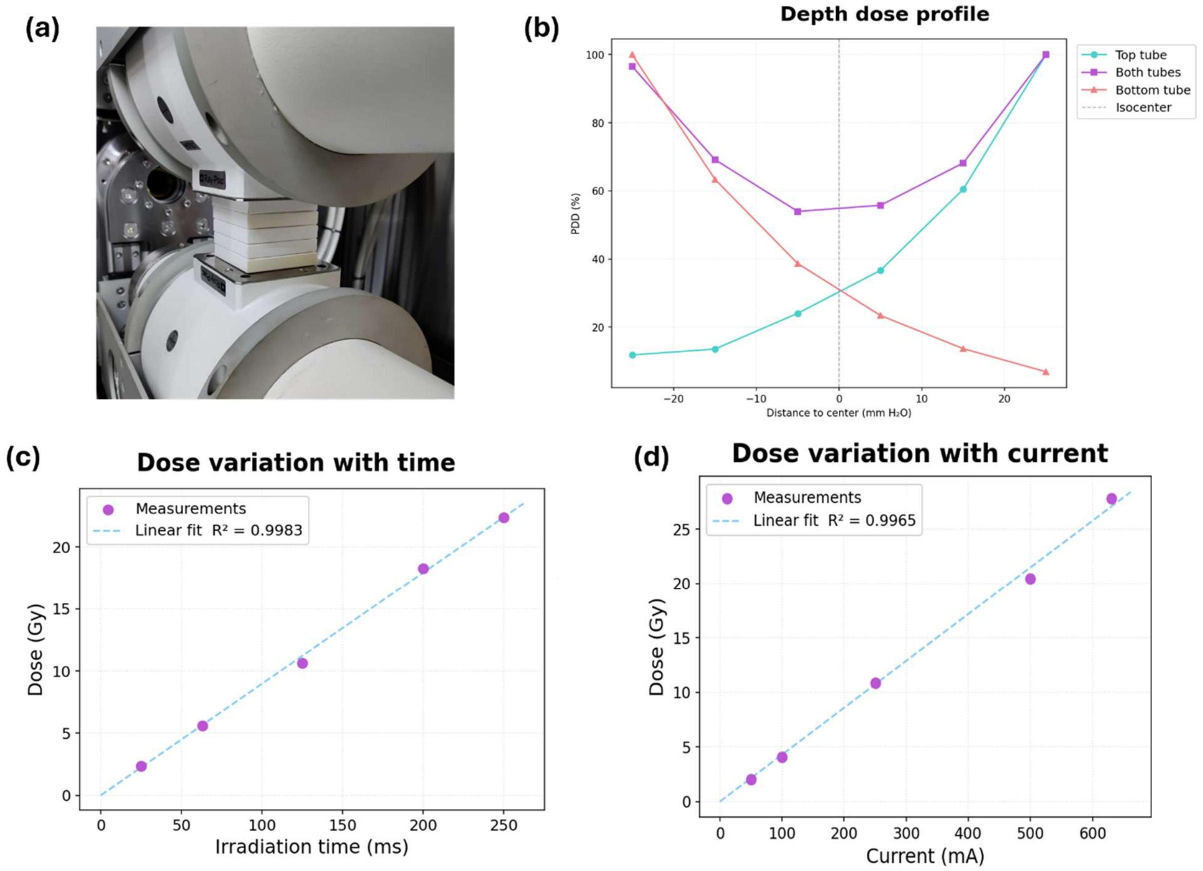

Supplementary Figure 1. Beam Characteristics. (a) A photo of the setup used to obtain the PDD. Gafchromic film was placed between the solid water slabs, as well as directly at the surface of both tubes. **(b)** Percentage Depth Dose (PDD) curves of irradiations performed with the top (blue), bottom (red), and both tubes (purple) in solid water slabs. **(c)** Linearity of the irradiation time, measured with Gafchromic films at SSD = 7 cm from both tubes. A linear fit is determined based on these measurements. **(d)** Linearity of the current, measured with Gafchromic film at SSD = 7 cm from both tubes.
